# Inclusiveness and Diversity in Citizen Science

**DOI:** 10.1007/978-3-030-58278-4_14

**Published:** 2020-08-29

**Authors:** Carole Paleco, Sabina García Peter, Nora Salas Seoane, Julia Kaufmann, Panagiota Argyri

**Affiliations:** 1 Museum für Naturkunde Berlin – Leibniz, Institute for Evolution and Biodiversity Science (MfN), Berlin, Germany; 2 Faculty of Science, Leiden University, Leiden, The Netherlands; 3 Earthwatch Europe, Oxford, UK; 4 Faculty of Geo-Information Science and Earth Observation (ITC), University of Twente, Enschede, The Netherlands; 5 OpenSystems, Departament de Física de la Matèria Condensada, Universitat de Barcelona, Barcelona, Spain; 6 Department of Applied Information Technology, University of Gothenburg, Gothenburg, Sweden; 7 Department of Bioscience Engineering, University of Antwerp, Antwerp, Belgium; 8 Museum für Naturkunde Berlin – Leibniz, Institute for Evolution and Biodiversity Science (MfN), Berlin, Germany; 9 Royal Belgian Institute of Natural Sciences, Brussels, Belgium; 10 Margherita von Brentano Center for Gender Studies, Freie Universität Berlin, Berlin, Germany; 11 Ibercivis Foundation, Zaragoza, Spain; 12 Museum für Naturkunde Berlin – Leibniz Institute for Evolution and Biodiversity Science (MfN), Berlin, Germany; 13 Evangeliki Model High School of Smyrna, Athens, Greece; 14 Science for Change, Barcelona, Spain

**Keywords:** Communities, Democratisation, Gender, Inclusion, Minority, Representation, Inclusiveness, Opportunities, Equal, Targets, ECSA, DITOs, D-NOSES

## Abstract

An ‘inclusive citizen science’ practice encourages engagement from all members of society, whatever their social status, sociocultural origin, gender, religious affiliation, literacy level, or age. In this chapter we will first address the question of inclusiveness in citizen science and how this is tackled. We will analyse the current situation of a number of projects and initiatives within the Citizen Science COST Action CA15212 and the Horizon 2020 SwafS programme, examine the data, and discuss the main factors that encourage or hinder inclusiveness. We will offer recommendations for a possible plural participation in citizen science activities and reflect on how research is improved when diverse citizens are used as in-the-field experts. We will demonstrate how research questions can be fine-tuned and how research impacts are enhanced through citizen participation, with a focus on gender representation. Bottlenecks can occur when considering inclusiveness in citizen science, including in data interpretation, tasks that require long-term participation, and tasks that have specific language and intermediation requirements.

## Introduction

Citizen science is a way to democratise science by including diverse groups of participants in the different stages of the research process (Hecker et al. 2018). It provides a particularly striking opportunity to rethink questions of inclusiveness in knowledge production: ‘citizen science poses questions about who participates in science, what it means to participate in science, who gets to decide what scientific questions to investigate, and even what kind of knowledge and practice count as science’ (Pandya et al. 2018). The aspirations and advantages of many citizen science initiatives are *openness*, *accessibility*, and *citizen-driven participation* (Fiske et al. 2019). Through the introduction of new and diverse groups to the scientific community, new perspectives on research questions, interpretations, and methods can develop (Bang et al. 2007, in Pandya 2012). Studies have shown that *diversity* benefits all learners, not just those from minority communities (Gurin et al. 1999, in Pandya 2012). Bonney et al. (2016, p. 12) conclude that ‘if the field of citizen science is to truly contribute to democratizing science, then it must strive to reach a wider range of audiences and participants’. This is why *inclusiveness* (in terms of participation) is a core part of citizen science and should be examined along different axes such as gender, ethnicity, socio-economic and sociocultural status, location, and educational level, alongside how these axes intersect to define hierarchies and power relations. For this, an intersectional perspective can be useful (see Okune et al. (2018)). More specifically, with regard to gender, different organisations have developed in their toolkits and principles (ECSA 2015)^1^ good practices for balancing the composition of citizen science teams and ensuring that women assume leadership roles in citizen science projects (Puy and Angelaki 2019).

This chapter introduces inclusiveness approaches and trends developed in different international contexts and then leads to three subsections that focus on inclusiveness more particularly within the EU research framework programmes, tackling policies, projects, and practices, including equal opportunities and gender representation within COST Action CA15212 *Citizen Science to Promote Creativity, Scientific Literacy, and Innovation throughout Europe*^2^ and citizen science projects. The chapter demonstrates the added value and improvements that inclusiveness can bring to citizen science projects and research. To conclude, recommendations, challenges, and future trends in this area are addressed.

## Inclusiveness in Citizen Science: Gaps and Trends

In this section, we will identify the many different profiles of participants involved in citizen science activities and then outline the most important developments in the evolution of inclusiveness in citizen science so far. In addition to general reflections on participation (Land-Zandstra et al., this volume, Chap. 10.1007/978-3-030-58278-4_13), this chapter adds insights on diversity issues among participants and volunteers. There has not yet been a nuanced, detailed analysis of who participates in citizen science activities (Haklay and Francis 2018), or a formal meta-analysis of representation in citizen science (Pandya et al. 2018). Only a few analyses have been undertaken that emphasise the different demographic characteristics of participant volunteers, mostly in the US and UK contexts. Some of them are summarised below:

- Pandya et al. (2018, p. 159) suggest that ‘participation in citizen science, at least in the United States, does not reflect the demographics of the population, and that this schism hurts both citizen science and underrepresented groups. Individuals from groups that have been historically underrepresented in science (e.g. African Americans, Latinos, American Indians) participate less than majority groups and affluent participants outnumber less-affluent participants’. The US National Academies of Sciences, Engineering, and Medicine in an analysis of training camps for volunteer and field experience, also indicate the over-representation of generally older white females with above average education levels (Pandya et al. 2018, p. 160; Frensley et al. 2017, p. 3).
- In the online US citizen science aggregator platform SciStarter 2.0, the majority of 653 SciStarter profiles completed by the end of 2017 were female (64%) in the 35–44 age range (female median, 41; male, 47) (Pandya et al. 2018, p. 160).
- In biodiversity citizen science projects (Theobald et al. 2015; Burgess et al. 2017), 125 of the demographic profiles of participants in 329 projects were white (88.6%), while 6.1% were Hispanic and 4.6% were Asian, including Asian Americans, while Wright et al. (2015), in their study of the Second Southern African Bird Atlas Project, found that volunteers were overwhelmingly older white males with high levels of education and income.
- In two ornithology citizen science projects in the UK, studied by Edwards et al. (2018), 83% of respondents were male, and 67% of respondents had a university-level qualification. However, the links between volunteers’ prior level of educational qualifications and disciplines studied are not uniform across citizen science projects.
- A report by OPAL^3^ showed parity in terms of participants’ gender (51% female). The number of non-white participants was also relatively high (23% in comparison with the total population in the UK of 16% non-white UK or Irish). People with disabilities, however, were fewer: only 9% of the participants, compared to 18% of the total population.
- Groups, such as low-income people, people with disabilities, and people of colour, are traditionally under-represented in environmental volunteering (Ockenden 2007).
- Most surveys show who are more highly qualified and from higher socio-economic backgrounds are most likely to participate as volunteers in citizen science projects (e.g. Garibay Group 2015).

Due to these reported trends, specific actions and efforts are needed to expand the diversity of participants in citizen science projects. As projects in citizen science grow, the number of volunteers will increase in turn. However, there should be a major research interest in the motivations of voluntary participation if we take into account different axes of discrimination. Just as motivations differ between individuals, they also may differ for the same person at different times (Clary et al. 1992; Ryan et al. 2001). In other words, it is necessary to understand the cultural, social, economic, and natural barriers that currently stand in the way of volunteering involvement (Roy et al. 2012). Using inclusive approaches, which are at the core of the citizen science movement, could be a solution. There is already an observable shift in the field from the focus on participation per se to the importance of inclusive participation.

It is proposed that encouraging more diversity of participants in citizen science projects will benefit scientific outcomes by delivering them to a wider population and growing *science capital* (Edwards et al. 2018). One evolution that can be observed in this area is that more communities are devising and leading their own citizen science projects (Ballard et al. 2018; Mahr et al. 2018) providing practitioners the opportunity to support grassroots community involvement throughout the research process. This has brought with it new trends, for example, the organisation of ThinkCamp events to harness the potential of creative collaboration and support inclusive, co-creation approaches to citizen science (Gold and Ochu 2018).

At the European level, in 2018, the European Citizen Science Association (ECSA) set up a working group – Empowerment, Inclusiveness and Equity^4^ – to establish collaborations with other approaches as *community-based research* (CBR), *transdisciplinary research*, and *participatory action research*. The goal is that more people from diverse backgrounds can participate in citizen science and other activities with collaborative approaches, shape them according to their wishes, and generate impacts that address their needs.

## Inclusive Approaches in European Commission Research Initiatives

Inclusiveness is one of the principles that guide the European Commission’s (EC) work. In recent years, the EC has intensified the consultation process with stakeholders that benefit from the research programme funding, inviting them to take part in the drafting of these work programmes.

While citizen science is already linked to multiple organisations, grassroots groups, and associations (Göbel et al., this volume, Chap. 10.1007/978-3-030-58278-4_17), an inclusive approach has been developed within a number of European Union research programme initiatives. In this section, we will outline three case studies to show how inclusiveness can be addressed, starting with the COST Action Programme.

When funding agencies started to include citizens as stakeholders within projects, the added value of citizen science was emphasised, and the involvement of citizens through citizen science activities increased. The ‘Science with and for Society’ (SwafS) programme helped integrate citizen science policies within the EC research funding mechanisms, although they are now mainstreamed in the *open science* activities through the Horizon 2020 programme.^5^ Two of the main EU projects funded under the SwafS call supporting citizen science are D-NOSES and DITOs. The first proposes a model to tackle inclusiveness within stakeholder engagement, and the second achieves deep public engagement in science and technology in Europe through the implementation of innovative and inclusive participatory events. We will review both projects.

### Multifaceted Inclusiveness in the COST Action Programme

The COST Action Programme^6^ has developed an *inclusiveness policy* around three main elements: geographical spread, career stage (involving early career investigators), and gender balance.^7^ The geographical spread is focused on less research-intensive countries, termed Inclusiveness Target Countries (ITCs) or widening countries. According to ERDYN Consultants and the Centre for Social Innovation (ZSI) recent impact assessment study, respondents from ITCs appear to receive greater career impact from COST Actions than their non-ITC colleagues (Knecht et al. 2019). They also notably benefit from the fact that COST Actions usually have larger consortiums (9.1% added value for ITC, compared to 2.9% for non-ITC) than other programmes and that COST meetings are held more regularly. The respondents also confirmed a change to research networks via COST Actions – they expanded in general and specifically included significantly more ITC participation (Knecht et al. 2019).

Cost Action CA15212, which will be examined in detail below, aims to harness research capacity across Europe to investigate and extend the impact of the scientific, educational, policy, and civic outcomes of citizen science with stakeholders from all sectors concerned (e.g. policymakers, social innovators, citizens, cultural organisations, researchers, charities, and non-governmental organisations). The goal is to gauge the potential of citizen science as an enabler of social innovation and socioecological transition. In total, 37 countries participated in this Action – 20 were characterised as ITC (54%). This is reflected in the Management Committee (MC), where 37 out of 68 MC members were from ITCs (54%).

In terms of gender, there was a balance with 35 female and 34 male MC members. The distribution of gender within ITC members is also well balanced (Table 14.1).

**Table 14.1:** Distribution of MC members from ITCs and their gender

ITC	Female	Male
Yes	19	18
No	16	16
**Total**	**35**	**34**

Source: Cost Action CA15212, 27.4.2020

Cost Action CA15212 had a policy to include all European countries and developed some special tools for ITC members to increase inclusiveness. One important measure was to run workshops, training schools, and MC meetings in ITCs. This helped to increase the number of participants from these countries and the opportunity for local stakeholders to participate.

It is not easy to determine the configuration of the active Cost Action CA15212 community. For example, some members of the MC do not attend meetings. Others are very active, but have no formal role, or self-fund their participation in workshops and are therefore more difficult to track administratively. Up to April 2020, 795 participants had contributed to 50 workshops, training schools, and MC meetings (Table 14.2). While at the MC meetings (the key annual meeting and decision-making forum) around 40% ITC participants were represented, at the workshops the number decreased to 25%. The percentage of female participants was about 50% but differed depending on the topic. Events that were linked to the social sciences were dominated by females, while events dealing with more technical aspects, such as data quality, data standards, and ontology models, typically had more male participants (Table 14.3).

**Table 14.2:** Number of events, event type, and number of participants by gender and ITC (data from 2017 to April 2020)

Number of events	Event type	Number of participants	Number of female participants	Percentage of female participants	Number of ITC participants	Percentage of ITC participants
5	MC meeting	209	106	50.72%	86	41.15%
4	Training school	78	38	48.72%	29	37.18%
41	Workshop	512	265	51.76%	128	25.00%
**50**	**Total**	**799**	**409**	**51.19%**	**243**	**30.41%**

**Table 14.3:** Percentage of female participants by event topic

Event topic	Percentage		
	Females	ITC	n=
Concepts and Methodological Framework for Mapping Stakeholders in Citizen Science	100.00%	57.14%	7
Citizen Science and Gender	100.00%	28.57%	7
Citizen Science & Social Innovations	81.82%	63.64%	11
Citizen Science Training School Barcelona	80.00%	40.00%	10
People-Places-Stories	77.78%	0.00%	9
Citizen Science in Social Sciences and Humanities	76.92%	30.77%	13
Co-creating the European Citizen Science Platform of the Future	73.68%	36.84%	19
Citizen Science in Social Sciences and Humanities	73.33%	33.33%	15
Citizen Science Strategies in Europe	70.00%	10.00%	10
Synergies of Citizen Science and Education	69.23%	30.77%	13
Progress and Prospects of Exploring Synergies between Citizen Science and Education	66.67%	11.11%	9
Doing Better Citizen Science ‘From Data Quality to Project Design’	65.38%	30.77%	26
Exploring the Interplay between Human Learning and Machine Learning	64.29%	21.43%	14
Motivation of Participants in Citizen Science Projects	63.64%	18.18%	11
Citizen Science Strategies in Europe – MC Meeting	61.40%	50.88%	57
Citizen Science Training School Erice	58.33%	20.83%	24
City + Citizen Science	58.33%	41.67%	12
Develop Concepts for Training Workshops to Enhance Synergies between Citizen Science and Education	57.89%	21.05%	19
Vespucci Training School on Digital Transformations in Citizen Science and Social Innovation	56.00%	32.00%	25
Systematic Review on Training Requirements and Recommendations	55.56%	22.22%	9
A pan-European Comparison of the Development and Implementation of CS Strategies / Policies	55.56%	22.22%	9
Building a Community Network on Synergies between Citizen Science and Education	53.85%	23.08%	13
Fourth Citizen Science Cost Action – MC Meeting	50.00%	34.48%	58
Degrees of Public Participation in Scientific Research	50.00%	58.33%	12
Recommendations for the Development of (national) Citizen Science Strategies	50.00%	45.00%	20
Author Meeign	47.37%	13.16%	38
Citizen Science and Open Data: A Model for Invasive Alien Species in EU	47.37%	0.00%	19
Roadmap to Consolidate and Expand the Knowledge Base on Participation and Learning in Citizen Science	47.06%	0.00%	17
Third Citizen Science Cost Action – MC Meeting	45.71%	45.71%	35
Citizen Science and Environmental Monitoring	45.45%	36.36%	11
Kick – it – off – the – ground – MC Meeting	44.07%	35.59%	59
Lessons Learned from Volunteers’ Interactions with Geographic Citizen Science Applications	41.67%	41.67%	12
Citizen Science and Open Science	41.67%	0.00%	12
Develop and Test an Ontology for Citizen-Science Metadata	35.71%	7.14%	14
Citizen Science Social Innovation as Promoter of RRI	33.33%	55.56%	9
Identifying and Describing Major Challenges for Citizen Science in the Next Decade	33.33%	16.67%	6
On Citizen-Science Ontology, Standards and Data	30.00%	5.00%	20
Creating a Citizens’ Information Pack on Ethical and Legal Issues around ICTs	35.29%	17.65%	17
Ensuring scientific quality of Citizen Science through data quality and project design	28.57%	14.29%	14
Citizen Science as a Tool for Education / Promotion of Scientific Literacy in Evolution	28.57%	14.29%	7
Quality Aspects in Citizen Science	25.00%	65.5%	8
Inclusiveness and Equal Opportunities in Wikipedia Publishing	25.00%	37.5%	8
Towards a New Ontology of Citizen Science	22.22%	33.33%	9
Coordination of Efforts with Existing Networks and Groups Working on Standardization in Citizen Science	16.67%	50.00%	6
Citizen Science Training in Coimbra	10.53%	63.16%	19
House of Apps: Create great apps for citizens	0.00%	0.00%	6

Another gender-related aspect is the impact of Cost Action CA15212 on both female and male participants. Knecht et al. (2019) highlight that the positive impact is more indirect for female participants, as they strengthen their reputation by participating in Cost Action activities. This would be a strong argument for women’s participation. Since many are held back in their careers by personal choices made to maintain the family-work balance, they could compensate by being active player in COST Actions. The greatest added value is seen by female participants in personal development, such as increased self-confidence and knowledge acquisition, which male participants did not report.

Under Cost Action CA15212, two workshops were organised specifically to increase inclusiveness: the first on Citizen Science and Gender^8^ and the second on Inclusiveness in Wikipedia Publishing.^9^ Both workshops have highlighted women’s under-representation in (citizen) science. The first one, in March 2019, did so through the experience of female scientists in Romania who presented their countless efforts in engaging young females in science through citizen science camps and acting as role models.^10^ The second workshop took place in Brussels, in March 2020, with trainer Daniëlle Jansen, an expert in Wikimedia projects (Wikipedia, Wikidata, and Wikimedia Commons), inclusiveness, and gender, who encourages female citizen scientists to publish on Wikipedia, and Quentin Groom from Meise Botanic Garden who works on the use of information technology in the analysis and dissemination of scientific information (see Box 14.1).

Cost Action CA15212 workshops have also demonstrated that providing networking opportunities can help to overcome knowledge gaps due to gender, educational level, and geography. A key challenge is to give value to what non-scientists have to share and encourage them through training sessions and meetings with scientists to adjust their level of involvement depending on their current resources, as proposed by the DITOs Escalator Model (DITOs 2019) (see the Recommendations section).

Achieving inclusiveness and diversity in citizen science projects needs a collaborative environment that provides learning and development opportunities in order to ensure the quality of research. Evaluation criteria of gender and diversity aspects in citizen science projects are also required.

### The D-NOSES Inclusive Engagement Model

D-NOSES^11^ is an ambitious citizen science project, funded by Horizon 2020, which aims to include *odour pollution* in policy agendas worldwide. D-NOSES is committed to being inclusive in the *citizen engagement* process. This includes people from different sociocultural backgrounds, socio-economic status, literacy levels, religious affiliations, minority groups, gender, age, people with disabilities, etc. They share the common issue of being affected by odour problems in their communities. Odour pollution is the second largest category of environmental complaints globally (ADEME 2005) even though it is an under-regulated issue that leaves citizens and entire communities unprotected and often leads to socio-environmental conflict. D-NOSES has developed an innovative methodology to improve odour issues at the local level using citizen science and participatory mapping strategies –

#### Box 14.1: Workshop on Citizen Science and Inclusiveness in Wikipedia Publishing

With over 134,000 active contributors on the English-language Wikipedia alone, it can be argued that Wikipedia is the most diverse international citizen science project in terms of usage, participants, and languages. It has an irreplaceable role in formal and informal education and in the democratisation of information globally. Furthermore, since 2012, Wikimedia has developed Wikidata with multilingual, public domain data. The workshop helped to identify the knowledge gaps that prevent some population groups from using these tools, most notably female citizen scientists. Women are indeed under-represented on Wikimedia – for example, only 11% of women publish on the Dutch-language pages and 20% on the English-language pages. Women account for only 17% of the biographies published on the English-language Wikipedia pages. This is a major concern – the low level of female representation could be due to a lack of information, difficulty in accessing the tools, or other tangible obstacles that impair women from being involved and included in this community of practitioners. During the workshop, discussions explored various ways to encourage women to take part in the Wikimedia community. First, we need to avoid making rigid distinctions between female non-scientists and female scientists, and even male non-scientists and male scientists. Non-scientists and women are inhibited from publishing and think that it is not for them. This is observable in Europe and the USA. Daniëlle Jansen stressed, however, that it is very different in the Caribbean, where women networks are traditionally in charge of education and knowledge transfer. Thus, there is a lot to learn from practices in different geographical areas and cultures, notably including women and their networks that can leverage support. We need to disinhibit women and offer them training and promote the use of their local and national languages to publish their articles on Wikipedia. Recognising such inputs as being equally important as the articles published on the English-language pages will encourage under-represented communities to participate.

this is being validated in ten different pilot sites in Europe, Chile, and Uganda. The main tool for data collection to collaboratively build odour maps is the citizen science *open app* OdourCollect (odourcollect.eu). With the community maps platform Odours Affecting Communities, communities affected by odour issues can map them collaboratively so they can be viewed by all. All D-NOSES tools and resources are being placed in the project’s Odour Observatory (odourobservatory.eu), the first of its kind. The project enhances digital inclusiveness and science education regarding the use of new technologies, particularly for women and girls. The project has also created other tools for ensuring inclusiveness in specific social environments, for example, the Smell Diaries^12^ for the elderly or people with difficulties in accessing digital technologies. The D-NOSES methodology aims to empower citizens and key stakeholders to generate, access, and use data related to odour pollution. The collected data is then used to inform and co-design possible solutions to better manage and mitigate odour problems. Thus, stakeholder engagement – particularly citizen engagement – is fundamental to its model.

**Fig. 14.1:**
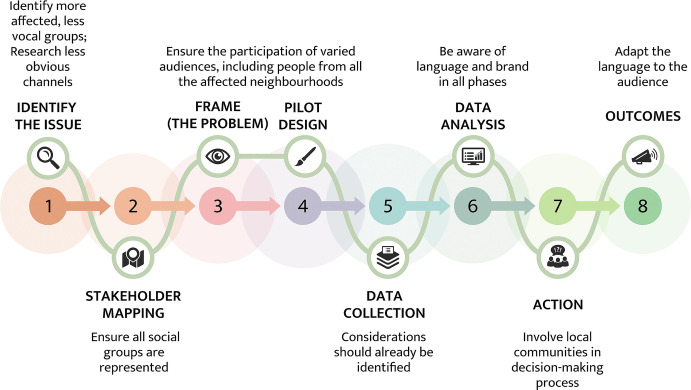
The D-NOSES inclusive engagement model The D-NOSES phases for the pilot case studies, plus the recommendations and tools to meet inclusiveness in stakeholder engagement in citizen science initiatives, have been co-created in the D-NOSES Consortium (particularly through partners Mapping for Change, Ideas for Change, and Ibercivis). Partners Mapping for Change and Ibercivis have benefitted from additional funding from two Short Term Scientific Missions under the COST Action CA15212 to work on the development of the D-NOSES engagement model, amongst other topics of interest

The project aims to engage the *quadruple helix* of stakeholders (*citizens and CSOs, public authorities, industry and SMEs, and academia*) while ensuring inclusiveness and diversity in engagement. Odour pollution may have similar effects in neighbourhoods with completely different socio-economic profiles. One of the key challenges is how to orchestrate the engagement of different stakeholders – citizens, CSOs and NGOs, industries, local and regional authorities, odour experts, etc. – as they can be affected in different ways by the problem and have conflicting interests and goals. The D-NOSES *engagement model* is based on engagement models from project partners Ideas for Change (the Bristol Approach, Rogers et al. 2017) and Mapping for Change (Haklay and Francis 2018). D-NOSES will combine best practices from both models and expand them with new methods and tools specific to the domain of odour pollution, the quadruple helix approach, and inclusiveness. The aim is to involve people from different social backgrounds in all the project phases – from *problem definition* to *pilot design* to *data collection*, including contributing to *action*, following the *extreme citizen science* approach (see phases in Fig. 14.1).

The phases of the D-NOSES inclusive engagement model are outlined in Fig. 14.1. Partners leading pilot case studies are encouraged at an early stage to understand the social realities of the areas affected by odour issues being focused on. In each of the phases, the project aims to identify the communities affected by odour pollution (engaging not only the ‘usual suspects’, i.e. people already interested in science, but all community members) and co-create methods and tools to engage them in the project and improve their quality of life. The model starts with desk research and then leads to fieldwork and ethnographic research. It includes key stakeholders to conduct preliminary conversations, to better understand the existing different realities. Co-creation workshops are a key method used to make people feel the project is theirs, contributing to their involvement as active actors who construct actions within the phases proposed, and eventually contribute to local decision-making. The pilot studies are shaped by the co-creation of the actors concerned. One of the main challenges of citizen science projects is to involve and engage participants who can contribute to data collection for a sustained period of time. Moreover, it is difficult to have a diverse group of people who may not be familiar with one another nor exposed to public participation in their locales. At the end of the chapter, recommendations and conclusions are made regarding how to meet the need for inclusiveness by following the D-NOSES engagement model.

### DITOs: Addressing Gender and Inclusiveness

With more than 3.8 million people online, the Doing It Together Science (DITOs) project reached an enormous number of participants. Events were organised in 18 countries – 15 EU member states, Switzerland, the USA, and Israel. Belgium hosted the largest number of events followed by the UK, Slovenia, and the Netherlands.

**Fig. 14.2:**
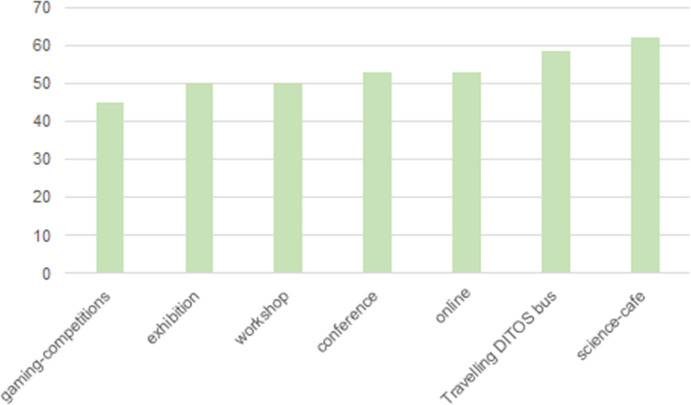
Female participation according to DITOs event type

Including workshops, science cafes, gaming competitions, and the travelling DITOs bus, more than half of the DITOs events (441/829) used interactive formats involving 165,372 citizens. DITOs events reached people of all ages. Those under the age of 20 and those aged 50–80 participated the most. In total, 48.5% of all DITOs event participants were female. The BioBlitzes and conferences had particularly strong female participation with 56.7% and 54.5%, respectively, while there was a higher percentage of male participation in game-related events (see Fig. 14.2).

For DITOs, it is interesting to note that gender participation did not depend on the event facilitator’s gender, nor did it vary much between event types. However, female participation varied significantly between different countries. DITOs results seem to be in line with other studies reporting difficulties in attracting women to science studies, for example, in German-speaking countries (Kröll 2010).

**Fig. 14.3:**
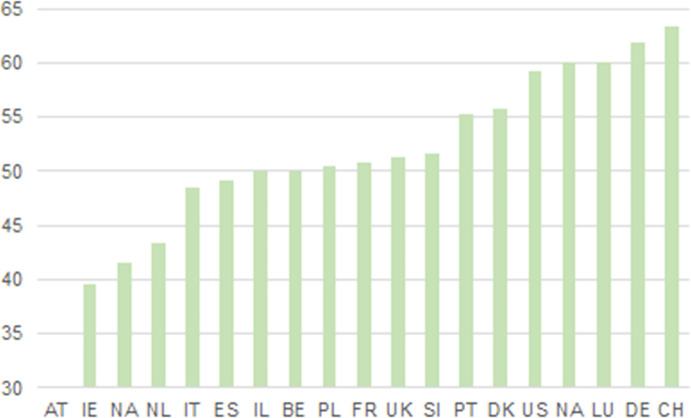
Percentage of female participation in events per country. No relevant data for Austrian policy round table in the reporting period. Note the scaling between 30% and 65%

All DITOs events went through an evaluation process to collect information on the participants’ profiles, including gender. In general, gender distribution was based on estimates from the event facilitators or organisers, but for some events gender information was based on participant questionnaire data. Note that Fig. 14.3 shows a relatively equal distribution between just under 40% and just over 60% females. The age axis has been scaled to emphasise any differences in gender participation.

Interestingly, higher percentages of female event participation come from Switzerland, Germany, and Luxembourg – where traditionally female STEM (science, technology, engineering, and mathematics) student rates are lower than the European average. This may be due to the fact that some of the activities in DITOs were about communicating scientific processes rather than producing science, thus encouraging citizens to engage in science. Such an approach may have been appealing to an audience that is not interested in STEM activities.

## Recommendations

As the definition of citizen science is contested (Haklay et al., this volume, Chap. 10.1007/978-3-030-58278-4_2), we recommend that citizen science is explained to *target audiences* before they start a project or activity. Indeed, from the evaluation of the practices described, for example, in the DITOs project, the partners highlighted that ‘creating inclusion begins within the organisation/team/facilitator making sense of the terms they are promoting and then designing events around that’ and that ‘inclusion means starting with the needs/interests of participants but that to be inclusive you need to be also exclusive’ (DITOs 2019). Inclusion is also about understanding and learning from the target audience. Citizen science and participatory science are often unfamiliar concepts for participants; project leaders may need several iterations of defining terms and objectives so that they are understandable and expectations can be made clear.

The term science in itself is sometimes a barrier, and all project terminology must be chosen carefully to make sure that practitioners and volunteers talk the same language and have the same understanding of the objective. Time commitment is also key to create trust and facilitate fruitful collaboration (Senabre et al., this volume, Chap. 10.1007/978-3-030-58278-4_11).

In the DITOs project, through the implementation of the *escalator model*, the organisers approached activities and events viewing participants not only as data collectors or passive consumers of science activities, but with the aim of achieving creative scientific skills, analytic work, and science-based citizen engagement. It is important to understand the escalator as a number of forms of interaction, which are suitable for different types of audiences and their interests and varying capabilities of organisations and facilitators. Not all participants want to move up the escalator, and not all organisations are interested in ‘educating’ participants to become autonomous researchers.

Offering multiple project entry points as well as multiple ways to participate at different levels of commitment are key to engaging new and diverse participants. This requires acknowledging that people have very different interests and motivations for engaging in citizen science. Real inclusion within citizen science is more likely to occur if issues are framed around participants’ values, focusing on local and tangible concerns, and if individuals believe their actions have impact (Whitmarsh et al. 2010). Framing research problems as local issues can help to engage individual citizens if they feel a sense of place attachment (Devine-Wright 2013). This requires reconsidering the role of different axes of inequality (e.g. gender). By providing an inclusive and integrative framework, different groups are supported to engage with specific topics. The citizen science inclusiveness and gender balance has not been considered so far as a research topic. In order to increase inclusiveness also in the area of gender equality, gender balance should be striven for in all phases of a citizen science project.

Another recommendation is addressed to funding organisations supporting more engagement from citizens in science: to consider more inclusive citizen science approaches to ensure that organisations, projects, and activities take advantage of the broadened connection inclusiveness brings to stakeholders and a more diversified audience for project research.

Looking at the inclusive engagement model proposed by the D-NOSES project and reflecting upon its implementation in a number of ongoing citizen science initiatives resulted in a number of recommendations to meet inclusiveness. First, it should be acknowledged that engagement, involvement, and active participation is extremely costly in terms of human resources and time commitment. Engagement needs to be maintained continuously over time. The more project leaders or facilitators participate in actions and are present in the communities affected, the better and the wider community engagement is. This needs to be considered if aiming to achieve greater engagement in a citizen science project, particularly regarding inclusiveness.

Moreover, it is important to plan engagement actions in each project phase to ensure inclusiveness from the outset. Deepening the knowledge on the social realities of the affected communities and undertaking ethnographic fieldwork prior to engagement have been crucial to ensuring inclusiveness. Acknowledging the participation of citizens from the beginning of a project is important to better understand the different realities and shapes research questions, methods, and tools for engagement (e.g. adapting D-NOSES to the contexts and needs of citizens affected by odour issues). Participants need to feel part of the project, and the usual gap between ‘us and them’ should be avoided. Questions that need to be answered include: Have less vocal groups been identified? Has it been ensured that all groups are represented when choosing the stakeholders to involve? How can we ensure participation within the different social realities represented? Are we involving communities when constructing the engagement methods and tools? When and where is it better to conduct rapid appraisals or co-creation workshops to ensure a wide variety of participants? Are data collection strategies adapted to the capabilities of the different communities involved? Are the voices of citizens and communities really being heard? Are they able to participate in local decision-making with quadruple helix stakeholders, allowing for a positive change?

As an example, in the Barcelona pilot case study in the D-NOSES project, varied socio-economic and sociocultural realities have historically been affected by the same odour issues in the east of the city, by the coastline, where several odour-emitting industries cohabit with a variety of communities – from a socially disadvantaged area to a newly refurbished neighbourhood by the sea. Getting a deeper understanding of these realities has been crucial to involve people in the project and apply different engagement methods, data collection strategies, and tools accordingly. Participation in community events has also been significant for engagement and inclusiveness. In these events, we have been able to co-create engagement strategies to be more inclusive and achieve broader participation with the support of the already participating citizens. Getting to know the community channels of participation – in this case, CSOs and neighbourhood associations – has been relevant to organise encounters and workshops. Adapting the language to local terms within D-NOSES actions has also been valuable. In this way, people feel the project is theirs – increasing the impact of its actions and achieving inclusiveness.

## Challenges and Future Trends

Thanks to digitalisation, citizen science is experiencing a revival. In recent years, hundreds of projects have been initiated, encouraging people from different backgrounds to participate in the collection, labelling, categorisation, and counting of different types of data. Digital platforms and tools have been developed to organise these different processes of participation (Skarlatidou et al. 2019) in innovative forms. Digital infrastructures can present both obstacles and opportunities for more diverse ways of undertaking citizen science through multiple ways of participation. Social groups that have been historically excluded from the hegemonic processes of production of knowledge remain excluded, and new inequalities emerge. Improved access channels are needed to link the potential brought by digitalisation potential with those from diverse, nontraditional, and excluded backgrounds to foster inclusion, empowerment, and emancipation.

As we live in the information revolution era, where technology plays a key role, citizen science approaches should consider training on the use of technologies and mobile applications to prevent knowledge gaps and achieve diverse participation. In order to overcome the language barrier for non-English speakers, software and interactive websites should enable participants to publish in and use their own language to share local or national concerns and knowledge.

Providing incentives and career opportunities for young citizen science researchers will help to attract new volunteers. Within this framework, developing close, cooperative relationships between universities and non-governmental organisations on citizen science will have significant advantages. The professional infrastructure of universities (access to technology, well-equipped libraries, specialised staff) and their scientific expertise (in fields such as statistics, information technology, legal and ethical knowledge, quality assessment, and communication) can provide open access to the public in citizen science and support sponsors in carrying out research projects in citizen science.

As citizen science movements grow, we can observe that some segments of the population are more inclined to take part than others due to their level of education, their geographic location, and the network or social environment they belong to. While broadening diversity is desirable to ensure varied contributions to science in both quantity and quality, it is important, however, when undertaking projects to ensure that the uniqueness and diversity of communities is respected and represented. Identification of target participant groups allows for more effective engagement strategies to be implemented, including tailored materials, communications, and training. Running small-scale trials or focus groups within target communities is a common method of assessing the effectiveness of engagement techniques and the suitability of materials and methodology (Tweddle et al. 2012). It is important to consider how topics and audiences impact engagement. Some studies suggest that locality is an important aspect of engagement with citizen science and acts as a catalyst for sustained engagement. Designing activities and projects that are grounded in local issues creates a captive audience and can maintain engagement for longer periods (Rotman et al. 2012). Pandya (2012) proposes a general framework to design citizen science projects that align with community priorities and increase inclusion. This framework involves five actions for citizen science project development and implementation: (1) aligning research and education with community priorities, (2) planning for co-management of the project, (3) engaging the community at every step, (4) incorporating multiple kinds of knowledge, and (5) disseminating results from the work widely (outside of scientific publication). Bonney et al. (2016) focus on Community Science Projects (CSP) as a type of public participation model within science defined by the nature of the activities in which their participants engage and its potential to engage a range of audiences that typically have not previously engaged with science. Such projects meet people where they are —geographically, intellectually, and in terms of their values, interests, families, and jobs.

## Conclusion

In this chapter, we have tried to present a variety of inclusive models that can be taken as best practices to increase citizen inclusion in (citizen) science and in societal challenges. Another societal challenge in which inclusion has played a major role has emerged while writing this chapter: the COVID-19 health crisis, which has highlighted inclusion through health concerns and the need for rapid reaction from several stakeholders including governance organisations, science, citizens, and industry. An efficient and responsive quadruple helix has not yet been put in place, and it is probable that more channels and direct links need to be developed to achieve a coordinated response. Efforts should be made to foster inclusiveness and equal opportunities within all fours areas of society (industry, science, citizens, policymakers) and to form the quadruple helix. If each stakeholder and community could open more doors, form collaborations, and leave aside preconceived ideas towards the other three, they would enrich solutions for local, national, and global scientific issues.

## Footnotes

1 An example can be found at https://www.rri-tools.eu/

2 http://www.cs-eu.net

3 https://www.opalexplorenature.org/reports-updates

4 https://ecsa.citizen-science.net/empowerment-inclusiveness-equity

5 https://ec.europa.eu/programmes/horizon2020/

6 https://www.cost.eu/

7 https://www.cost.eu/who-we-are/cost-strategy/excellence-and-inclusiveness/

8 https://cs-eu.net/events/internal/workshop-wg-4-wg-6-citizen-science-and-gender

9 https://cs-eu.net/events/internal/wg1-workshop-inclusiveness-and-equal-opportunities-wikipedia-publishing

10 Report WG4 & WG6 Citizen Science and Gender – Iasi (RO) 03/2019 https://cs-eu.net/sites/default/files/media/2019/05/Report_WG4_WG6Workshop_CitizenScience_and_Gender_20190320.pdf

11 D-NOSES (Grant Agreement No. 789315) is a Horizon 2020 project funded under the SwafS call. For more details: https://dnoses.eu/about-d-noses/

12 https://odourobservatory.org/wp-content/uploads/sites/2/2019/09/Smell-diary-template.pdf
